# High-resolution shock-capturing numerical simulations of three-phase immiscible fluids from the unsaturated to the saturated zone

**DOI:** 10.1038/s41598-021-83956-w

**Published:** 2021-03-04

**Authors:** Alessandra Feo, Fulvio Celico

**Affiliations:** 1 Department of Chemistry, Life Sciences and Environmental Sustainability, Parma University, Parco Area delle Scienze 157/A, 43124 Parma, Italy; 2 INFN gruppo collegato di Parma, Parco Area delle Scienze, 7/A, 43124 Parma, Italy

**Keywords:** Hydrology, Mathematics and computing, Physics

## Abstract

Numerical modeling of immiscible contaminant fluid flow in unsaturated and saturated porous aquifers is of great importance in many scientific fields to properly manage groundwater resources. We present a high-resolution numerical model that simulates three-phase immiscible fluid flow in both unsaturated and saturated zone in a porous aquifer. We use coupled conserved mass equations for each phase and study the dynamics of a multiphase fluid flow as a function of saturation, capillary pressure, permeability, and porosity of the different phases, initial and boundary conditions. To deal with the sharp front originated from the partial differential equations’ nonlinearity and accurately propagate the sharp front of the fluid component, we use a high-resolution shock-capturing method to treat discontinuities due to capillary pressure and permeabilities that depend on the saturation of the three different phases. The main approach to the problem’s numerical solution is based on (full) explicit evolution of the discretized (in-space) variables. Since explicit methods require the time step to be sufficiently small, this condition is very restrictive, particularly for long-time integrations. With the increased computational speed and capacity of today’s multicore computer, it is possible to simulate in detail contaminants’ fate flow using high-performance computing.

## Introduction

Modeling the dynamics of a contaminant’s fate, nonaqueous immiscible fluid, and/or water into an aquifer medium, immiscible fluid migration in the unsaturated soil/porous medium to the saturated zone is challenging. The governing equations are coupled Richard’s equation^1^ for each phase and are nonlinear partial differential equations (PDEs), based on Darcy’s Law and the mass and momentum conservation principles. Recent reviews on state-of-the-art numerical solutions on the Richards’ equation through the vadose zone and saturated zone can be found in^2–5^, and Ref.^6^ for a much broader application on modeling soil processes.

Because these equations are highly nonlinear, degenerate elliptic-parabolic PDEs^7^, with a possible dominating hyperbolic advection term (the effect of gravity), cannot be resolved analytically, except for very simplified one-dimensional cases^5,8^. Therefore, numerical approximations schemes such as Finite Difference Method (FDM), Finite Element Method (FEM), or mixed combinations^9^, among others, are used to simulate unsaturated–saturated flow models. Several methods were proposed to resolve convergence issues and reduce mass-balance errors^10,11^. Several papers show results on numerical simulations for transient saturated-unsaturated water flow^4,12–15^. In^9^ an improved method for treating the unsaturated zone and saturated water flow together, using an improved FDM and FEM mixed combination is presented. The horizontal direction of the governing equation is discretized using the FEM, while the FDM is used in the vertical direction.

The objective of this paper is to study the dynamics of transient regime three-dimensional (3D) three-phase of heterogeneous, anisotropic, immiscible fluids flow from the unsaturated to the saturated zone, where one of these phases is a nonaqueous phase liquid (NAPL) and investigate how this contaminant front propagates and how it is distributed in temporal and spatial small/large scales evolution.

Example multiphase fluids flow models for NAPL infiltration in both, a saturated-unsaturated zone can be found in^16,17^. The governing equations are described through Richards’ equation as a conservation equation for each phase in a porous medium. The nonlinear character of these PDEs is represented by the relationship between the permeability-saturation, which is responsible for creating sharp (shocks) front, and capillary pressure-saturation of each phase. The functional dependence of these relationships may vary strongly, depending on the (hydro)geological features, the composition of the fluids, and the combination of the phases present in the system. The nonlinearity of the coefficients in the equation that holds at the continuum level is no longer guaranteed at the discrete level and have shown global mass balance errors^18–20^.

Most of the discretized methods used up to now use implicit methods in which the time is calculated backward, instead of explicit methods, that are forward in time. The implicit methods produce a high number of linear equations that have to be solved by a computer and become computationally expensive. On the other hand, explicit methods give a very clean way to calculate the dependence on time. They were not much used in the past because the time step size has to be very small; otherwise the numerical solution could rapidly diverge. Considering the high nonlinearity of the governing equations, these methods have been put aside.

New solutions techniques are called to improve discretization methods and scheme both in space and time. Time discretization is of paramount importance to study variably saturated phenomena in the transient regime and analyze real-time water/contaminant flow and migrations since space and time discretization affect computational accuracy and convergence. With the increased computational speed and capacity of today’s multicore computers, GPUs’ progress accelerated computing, new powerful programming tools (mostly open source) capable of enhancing modeling tools and managing big data. It is a great and unique moment and opportunity to simulate in detail the contaminants’ fate for a variably saturated flow.

The evolution in time is performed using an explicit method rather than the most used, implicit one. Explicit methods like the forward Euler method are straightforward compared with the implicit method but require the time step size to be sufficiently small since they are ’conditionally stable.’ This condition is very restrictive, particularly for long-time integrations, and requires the use of High-Performance Computing (HPC). In contrast to the explicit method, the implicit methods such as the backward Euler method and the Crank-Nicholson methods are unconditionally stable but very expensive from the computational point of view and vastly used in the literature, see, for example, the implicit iteration IMPES^21^ for two-dimensional heterogeneous porous medium.

This paper presents a high-resolution three-dimensional (3D) numerical results using a new code (CactusHydro) that uses an explicit method called high-resolution shock-capturing (HRSC) flux conservative method^22^ to treat jump discontinuities of the parameters. For the time evolution, we use an explicit method, the forward Euler method, that has not been used very often in groundwater flow literature. The main approach to the problem’s numerical solution is based on (full) explicit evolution of the discretized (in-space) variables. This technique ensures the mass-conservation of the various components and accurately propagates the fluid component’s sharp front. These explicit methods are not too much expensive as the implicit ones, but instead, they need a very small-time step size. For that reason, it is also necessary to implement HPC and, thus, massively parallelization to treat large scale problems with small-time step size.

CactusHydro is based on the Cactus computational toolkit^23^, an open-source software framework for developing parallel HPC simulation codes. We check our code’s validity with two analytical examples, the inviscid Burgers’ equation and the Buckley–Leverett model , and a 2D numerical unsaturated–saturated water flow model^9^ together with the sand tank experimental data conducted in Ref.^24^ (see Supplementary Information). We then apply to a case where we show results of a contaminant, a nonaqueous phase liquid (NAPL) released from the vadose zone that goes downward to the saturated zone. Our results are obtained by running our code on a parallel system machine with a large number of processors.

## Flow governing equations and mathematical setup

The equation that describes a multiphase fluid flow in a porous medium is calculated using the conservation equation for the mass and momentum for each fluid phase. For three-phase fluid flow in terms of nonaqueous (n), water (w), and air (a) is given by

1 $$\begin{aligned} \frac{\partial }{\partial t} (\phi \rho _\alpha S_\alpha ) = - \frac{\partial }{\partial x^i}(\rho _\alpha u_\alpha ^i) + q_\alpha \, , \end{aligned}$$

where $\alpha = (n,w,a)$ and, Darcy’s velocity for each phase is given by,

2 $$\begin{aligned} u_\alpha ^i = -\frac{k_\alpha ^{ij}}{\mu _\alpha } \bigg (\frac{\partial p_\alpha }{\partial x^j} + \rho _\alpha g \frac{\partial z}{\partial x^j} \bigg ). \end{aligned}$$

Substituting Eq. (2) into Eq. (1) we get,

3 $$\begin{aligned} \frac{\partial }{\partial t} (\rho _\alpha \phi S_\alpha ) = \frac{\partial }{\partial x^i} \bigg [ \rho _\alpha \frac{k_\alpha ^{ij}}{\mu _\alpha } \bigg (\frac{\partial p_\alpha }{\partial x^j} + \rho _\alpha g \frac{\partial z}{\partial x^j} \bigg ) \bigg ] + q_\alpha , \end{aligned}$$

where $x^i=(x^1,x^2,x^3)$ are the spatial cartesian coordinates, and $x^0=t$ is the time coordinate, $k_{\alpha }^{ij}$ is the effective permeability tensor $[L^2]$, $\rho _{\alpha }$ is the density $[\frac{M}{L T^2}]$, $\mu _{\alpha }$ is the dynamics viscosity $[\frac{M}{L T}]$, $p_{\alpha }$ is the fluid pressure $[\frac{M}{T^2 L}]$, *g* is the gravitational acceleration $[\frac{L}{T^2}]$, *z* is the depth [*L*], $q_{\alpha }$ is the mass source/sink $[\frac{M}{L}]$, $\phi$ is the porosity, $S_{\alpha }$ is the dimensionless volumetric saturation which is the fraction of the total available volume (inside the porous media) occupied by that component. These equations include 16 dependent variables in the general case, $\rho _w, \rho _n, \rho _a, S_w, S_n, S_a, \phi , k_w, k_n, k_a, p_w, p_n, p_a, \mu _w, \mu _n, \mu _a$, and the three terms, $q_w, q_n, q_a$. As a consequence of this, we need 13 additional relationships to obtain a solution for the system (3). One relationship corresponds to the sum of the volumetric saturations equal to one,

4 $$\begin{aligned} S_w + S_n + S_a = 1. \end{aligned}$$

For a three-phase fluid flow, we need two capillary pressures. In our formulation, we used the capillary pressure for the air-water phase and the capillary pressure for the air-nonaqueous phase, respectively

5 $$\begin{aligned} {{ p_{caw} = (p_a - p_w), \qquad p_{can} = (p_a - p_n),}} \end{aligned}$$

indeed, $p_a$, $p_w$ and $p_n$ are not independent and we have that the capillary pressure of the nonaqueous-water phase is given by, $p_{cnw} = (p_n - p_w) = (p_{caw} - p_{can})$.

In this paper, we developed a general formulation for three-phases immiscible fluids flow . We consider nonzero pressure gradients (see Ref.^25,26^ where the air gradient pressure is assumed negligible). In general, the capillary pressure is a function of saturation. From Eq. (5) we have two relationships. The porosity $\phi$ is a function of the pressure (and is one relationship). Densities and viscosities are funcions of phase pressures (and correspond to six relationships). The effective permeability tensor can be written as $k_w^{ij} = k_{rw} \, k^{ij}$, $k_n^{ij} = k_{rn} \, k^{ij}$, $k_a^{ij} = k_{ra} \, k^{ij}$, where $k_{rw}, k_{rn}$, and $k_{ra}$ are the dimensionless relative permeabilities for the phases *w*, *n*, *a* respectively. $k^{ij}$ is the absolute permeability, that depends on the properties of the porous medium. They are function of saturations (and are three relationships).

Finally, we can rewrite Eq. (3) using the capillary pressures (5) and the relative permeabilities,

6 $$\begin{aligned} \frac{\partial }{\partial t} (\rho _w \phi S_w)&= \frac{\partial }{\partial x^i} \bigg [ \rho _w \frac{k_{rw}}{\mu _w} k^{ij} \bigg (\frac{\partial p_a}{\partial x^j} - \frac{\partial p_{caw}}{\partial x^j} + \rho _w g \frac{\partial z}{\partial x^j} \bigg ) \bigg ] + q_w, \nonumber \\ \frac{\partial }{\partial t} (\rho _n \phi S_n)&= \frac{\partial }{\partial x^i} \bigg [ \rho _n \frac{k_{rn}}{\mu _n} k^{ij} \bigg (\frac{\partial p_a}{\partial x^j} - \frac{\partial p_{can}}{\partial x^j} + \rho _n g \frac{\partial z}{\partial x^j} \bigg ) \bigg ] + q_n, \nonumber \\ \frac{\partial }{\partial t} (\rho _a \phi S_a)&= \frac{\partial }{\partial x^i} \bigg [ \rho _a \frac{k_{ra}}{\mu _a} k^{ij} \bigg (\frac{\partial p_a}{\partial x^j} + \rho _a g \frac{\partial z}{\partial x^j} \bigg ) \bigg ] + q_a, \end{aligned}$$

in terms of the variables $p_a, S_w, S_n$, and $S_a$.

The system of Eqs. (4, 6) need to specify the five functions, $k_{r\alpha }=k_{r\alpha }(S_a,S_n,S_w))$, $p_{can}=p_{can}(S_a,S_n,S_w)$, and $p_{caw}=p_{caw}(S_a,S_n,S_w)$. We will be back to this point at the end of this section. But it is worth noticing that choices of solutions correspond to the possible different porous medium. But the numerical solution and the method we apply here is not affected by any particular option.

To complete the mathematical description, we need the expressions for $q_{\alpha }$, the boundary conditions, and the initial conditions. Before doing that, let us write the rock compressibility $c_R$ as a function of the porosity and pressure,

7 $$\begin{aligned} c_R = \frac{1}{\phi } \frac{\partial \phi }{\partial p}, \end{aligned}$$

and consider the Taylor expansion up to order one in $c_R$ where we get a linear approximation for the porosity

8 $$\begin{aligned} \phi = \phi _0 [1 + c_R (p - p_0)]. \end{aligned}$$

Let us define the product of the porosity $\phi$ and the saturation for each phase as, $\sigma _w \equiv \phi S_w, \sigma _n \equiv \phi S_n, \sigma _a \equiv \phi S_a$, then Eqs. (4) and (8) can be written as,

9 $$\begin{aligned} \sigma _a + \sigma _n + \sigma _w = \phi _0 [1 + c_R (p - p_0)], \end{aligned}$$

where $\phi _0$ is the porosity at the reference pressure $p_0$, which we consider to be the atmospheric pressure. Now we can write the left-hand side of Eq. (8) as a function of the pressure,

10 $$\begin{aligned} \frac{\partial \phi }{\partial t} = \phi _0 c_R \frac{\partial p}{\partial t} \end{aligned}$$

where *p* will be associated to $p_a$ and the system of PDEs (6) becomes

11 $$\begin{aligned} \frac{\partial \sigma _a}{\partial t} + \frac{\partial \sigma _n}{\partial t} + \frac{\partial \sigma _w}{\partial t} = \phi _0 c_R \frac{\partial p}{\partial t}, \end{aligned}$$

and ($$\alpha =w,n,a$$):

12 $$\begin{aligned} \frac{\partial \sigma _{(\alpha )}}{\partial t}&= - \frac{\partial }{\partial x^i} \bigg [ F^i_{(\alpha )}(S_w,S_n,S_a,p) \bigg ] + \frac{\partial }{\partial x^i} \bigg [ Q^i_{(\alpha )}(S_w,S_n,S_a,p) \bigg ] \end{aligned}$$

where the

13 $$\begin{aligned} F^i_{(\alpha )}(S_w,S_n,S_a,p) = - \frac{k_{r(\alpha )}(S_w,S_n,S_a)}{\mu _{(\alpha )}} k^{ij} \bigg (\frac{\partial p}{\partial x^j} + \rho _{\alpha } g \frac{\partial z}{\partial x^j} \bigg ) \end{aligned}$$

do not depend on the spatial derivative of the saturation and the

14 $$\begin{aligned} Q^i_{(\alpha )}(S_w,S_n,S_a,p) = - \frac{k_{r(\alpha )}(S_w,S_n,S_a)}{\mu _{(\alpha )}} k^{ij} \frac{\partial p_{ca{(\alpha )}}(S_w,S_n,S_a)}{\partial x^j} \end{aligned}$$

depends on the spatial derivative of the saturation, where we assumed constant density–viscosity for each phase.

The PDEs system to be numerically resolved is composed by (11, 12) and variables $p, \sigma _w, \sigma _n, \sigma _a$, together with the functional form of the relative permeabilities (15) and capillary pressures (19) (see next subsection). We separated the right-hand side of the system (12) in advection (hyperbolic) PDEs, the one that also depends on the spatial derivative of the pressure *p* and the gravity *g* [Eq. (13)], and parabolic PDEs, the one proportional to the capillary pressures $p_c$ [Eq. (14)] in the variable saturation. These two pieces will be treated differently when we used numerical methods. The hyperbolic PDEs is responsible for the shock formation when the flow passes through a discontinuity and has to be treated using a mass-conservative numerical method.

### Permeabilities and capillary pressures model

Now that we have the complete system of three-phase flow equations, we need to define the functional form for the relative permeabilities and capillary pressures. The relative permeabilities for three phases has been extended from the two-phase expressions^27^ and are given by,

15 $$\begin{aligned} k_{rw}&= S_{ew}^{1/2} [1 - (1 - S_{ew}^{1/m})^m]^2, \nonumber \\ k_{ra}&= (1 - S_{et})^{1/2} (1 - S_{et}^{1/m})^{2m}, \nonumber \\ k_{rn}&= (S_{et} - S_{ew})^{1/2} [(1 - S_{ew}^{1/m})^m - (1 - S_{et}^{1/m})^m]^2, \end{aligned}$$

where the total effective liquid saturation, $S_{et}$, is defined as

16 $$\begin{aligned} S_{et} = \frac{S_w + S_n - S_{wir}}{1 - S_{wir}}, \end{aligned}$$

and $S_{wir}$ is the irreducible wetting phase saturation. For the capillary pressure, we use the van Genuchten model^19^ where the effective saturation, $S_e$, has the following form,

17 $$\begin{aligned} S_e = [1 + \alpha p_c^n]^{(1 - \frac{1}{n})}, \end{aligned}$$

and $\alpha$ and *n* are model parameters. Defining $m = 1 - \frac{1}{n}$, from which we get, $n = \frac{1}{1 - m}$, and resolving for $p_c$ we obtain

18 $$\begin{aligned} p_c = - p_{c0} (1 - S_e^{1/m})^{1 - m}, \end{aligned}$$

where $p_{c0} = \alpha ^{-1}$ is the capillary pressure at $S_e=0$. The water content $S_w$ is entirely determined by the capillary pressure between the NAPL and water, $p_{cnw} = p_{cnw}(S_w)$. The total liquid content $S_t = S_w + S_n$ (or alternatively, the air saturation $S_a = 1 - S_t$) is completely determined by the capillary pressure between air and NAPL, $p_{can} = p_{can}(S_t)$. From the definition of capillary pressure, it follows that the capillary pressure between air and water is not an independent quantity but is given by, $p_{caw} = p_{can} + p_{cnw}$. As a consequence of these wettability assumptions, the local saturated distribution of a three-phase water-NAPL-air system is determined by two-phase capillary pressure-saturation relationships for air-water and air-NAPL. The capillary pressures are given by,

19 $$\begin{aligned} p_{can}&= - p_{can0} (1 - S_{et}^{1/m})^{1-m}, \nonumber \\ p_{caw}&= - p_{can0} (1 - S_{et}^{1/m})^{1-m} - p_{cnw0} (1 - S_{ew}^{1/m})^{1-m}. \end{aligned}$$

## High performance computing and CactusHydro

Most of the multiphase/three-phase groundwater flow equation solvers employ either FDM or finite element method (FEM) in the three coordinate space. Hybrid combinations are also possible^9^. Other used methods are: transversal methods of lines for the numerical modeling of vertical infiltration into the vadose zone^28,29^, or a WENO based method of lines^30^. Although there were very few attempts to use a supercomputer to simulate subsurface solute transport, the situation has changed in the last ten years.. New codes use HPC, for example, PFLOTRAN^31,32^, Parflow Hydrologic model^33,34^, RichardsFOAM^35^, CATHY (CATchment HYdrology)^36^. But it should be noticed that none of them systematically use explicit forward in time evolutions and conservative high-resolution shock capturing methods to efficiently study the dynamics of multicomponent flow in porous soil.

Most of these codes are written in Fortran language (as, for example, PFLOTRAN), while few are written in C++/C. Both programming languages are efficient for large computation problems and are well supported compared with other languages. Some codes, as for example, RichardsFOAM, only treat Richard’s equation and study the water fluxes at the watershed scale. Most of the codes used FDM, FEM, and less often, FVM. Some codes are commercial software such as, COMSOL Multiphysics^37^, FEFLOW (Finite Element subsurface FLOW system)^38^. It is expected that, if properly applied, different spatial discretization methods should not change results too much when other numerical methods are used.

Our interest is focused on the application of software and codes to large-scale hydrological modeling, similar to CATHY, ParFlow, PFLOTRAN, etc., and use sufficiently finer mesh size, especially in the vertical direction or refinement in the area where the contaminant or the principal problem is evolved. This paper presents a new code (CactusHydro) that performs high-resolution numerical simulations in multiphase flow from the vadose zone to the saturated one in a porous medium. CactusHydro is a code written in C/Python language and is based on the open-source Cactus toolkit^20,23,39^, an open-source software framework for developing parallel high-performance simulations codes. Data are evolved on a cartesian mesh with six refinement level using Carpet^40,41^. We discretize the domain problem using the FDM and implement the explicit (forward) Euler method for the space-time evolution of the system.

## Numerical methods

The strong nonlinearities in the previous system of PDEs are represented by the relationship between the permeability-saturation, which is responsible for creating the shocks, and capillary pressure-saturation of each phase. This situation has to be described accurately in time (and space) and needs high-resolution and refinement at a small scale (vertical and horizontal) up to a large scale. The crucial part to notice is the fact that the dominant piece of the multiphase flow PDEs that has to do with the water and nonaqueous phase is dominated by the hyperbolic part (the one proportional to gravity and the gradient of the pressure, rather than the elliptic part of the equation (proportional to the capillary pressure). We will use explicit methods to resolve the part of the PDEs equation that has a hyperbolic structure and resolve it in an explicit way to eliminate the oscillations/shocks that are caused due to the term proportional to the gravity constant.

When introducing the formal properties of numerical solutions methods of PDEs associated with conservation laws, two fundamental theorems are underlining the importance of using a conservative formulation. The first one is due to Lax, and Wendorff^42^ and the second by Hou and LeFloch^43^. Namely, that conservative numerical schemes, if convergent, do converge to the weak solution of the problem. The second theorem states that non-conservative numerical schemes do not converge to the correct solution if a shock wave (or discontinuity) is present in the flow. These two theorems state that if a conservative formulation is used, then we are guaranteed that the numerical solution will converge to the correct one, while if a conservative formulation is not used, we are guaranteed to converge to the incorrect solution in the likely event in which the flow develops a discontinuity.

The main approach to the numerical solution of the problem is based on a class of semi-discrete methods (Ordinary differential equation (ODE) in time and discretized in space) conservative flux methods of the type discussed in Kurganov and Tadmor (KT)^22^ using upwind fluxes. This approach is based on (full) explicit in the time evolution of the discretized equation using the so-called methods of lines (MoL) for the time evolution of the discretized (in space) variables. This technique allows to overcome the major limitation of the solution methods used before and to ensure mass conservation of the various components easily and accurately propagate the sharp front of the fluid component. This has the main drawback that the size of the time step that must be used to ensure convergence is much smaller than the one that can be used with an implicit scheme or semi-implicit one, like the one used in IMPES simulations^21^. In particular, to deal with the sharp front originated from the nonlinearity of the equations, we use a high-resolution shock-capturing (HRSC) method to treat discontinuities due to the capillary pressure (air-nonaqueous), (water-nonaqueous) and the permeabilities that depend on the saturation of the three different phases.

The conservation form (second-order scheme) in the 1D-case (straightforwardly generalized to 2D and 3D) is given by^22^,

20 $$\begin{aligned} \frac{d}{dt} u_j(t) = - \frac{H_{j + 1/2}(t) - H_{j-1/2}(t)}{\Delta x} + \frac{P_{j + 1/2}(t) - P_{j-1/2}(t)}{\Delta x} \end{aligned}$$

with the numerical flux,

21 $$\begin{aligned} H_{j+1/2}(t)= & {}\, - \frac{1}{2}\left[ F(u_{j + 1/2}^+(t)) + F(u_{j+1/2}^-(t))\right] - \frac{1}{2} a_{j+1/2}(t)\left[ u_{j+1/2}^+(t) - u_{j+1/2(t)}^-(t)\right] \end{aligned}$$

22 $$\begin{aligned} P_{j + 1/2}(t)= & {}\, \frac{1}{2}\left[ Q(u_{j}(t),\frac{u_{j+1}(t)-u_{j}(t)}{\Delta x} ) + Q(u_{j+1}(t),\frac{u_{j+1}(t)-u_{j}(t)}{\Delta x} ) \right] \end{aligned}$$

and the discretized variable $u_j(t)$ are approximated TVD limited piecewise linear (discontinuous at the cell interface) that assume the values $u_{j + 1/2}^-$ and $u_{j + 1/2}^+$ at the boundary between the cell *j* and $j+1$ and $a_{j+1/2}(t)$ is the maximum of the spectral radius of $F'(u)$. We can now use the crucial simplification (that it is now accurate at first-order in space) that holds true when *F*(*u*) does not change sign (as it is for our equations) for different values of *u*(*t*) at the interface. Namely we assume: $H_{j+1/2}(t) = - F(u_{j + 1/2}^+(t))$ if $F(u(t)) < 0$ and $H_{j+1/2}(t) = F(u_{j + 1/2}^-(t))$ if $F(u(t)) > 0$.

The HRSC methods possess the following properties: sharp resolution of discontinuities without considerable smearing; at least second-order of accuracy on smooth parts of the solution; absence of spurious oscillations in the solution; convergence to the “weak” solution as the grid is refined; no use of artificial-viscosity terms. The method belongs to the class of Monotonic Upstream-centered Scheme for Conservation Law (MUSCL) suggested by van Leer in 1973, and the KT scheme is second-order accurate in space. To achieve second-order accuracy, one needs to use analytical information on the form of the flux. However, as in this work, the use of the first-order upwind formula for the fluxes, and the minmod flux limiter, only the point values of the flux are required. This is of great advantage since it can use tabulated values for permeabilities. The only penalty is that it is first-order not only at the discontinuities but also over the whole simulation grid. But one has to keep in mind that any methods will be first-order at the physical discontinuities, and the problems we target to study involve following real-discontinuities.

## Results

In this study, some tests are performed to verify the accuracy and reliability of the HRSC method and the CactusHydro code. The results are compared with analytical models and experimental data results.

### The inviscid Burgers’ equation

**Figure 1:**
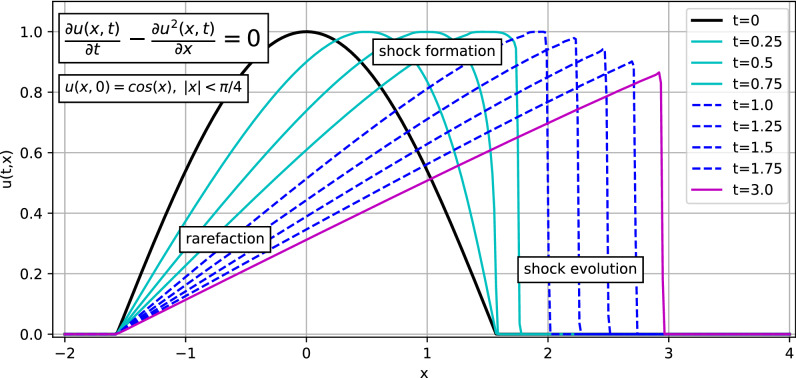
Formation of a shock and rarefaction using the Burgers’ equation. The solution at the initial condition for $t=0$ is represented by a cosine function moving from the left to the right and its time evolution using the HRSC FD method. Notice the formation of a discontinuity jump (shock) and the creation of a rarefaction.

To show the effectiveness of the method, we consider the inviscid Burgers’ hyperbolic equation, which represents a model for nonlinear wave propagation and how the use of a (simplified) HRSC method is capable of dealing with sharp fronts (or discontinuities) and to evolve them correctly as well as to follow the shock-formation and the rarefaction. Figure 1 shows (in black) the initial conditions $u(x,0) = cos(x)$ for $|x| < \pi /4$ and its evolution, moving from the left to the right, using the employed HRSC method at different values of *t* (colored lines). One may observe the formation of the jump discontinuity (shock). It is also observed the formation of a rarefaction when the propagation velocity ($v(x,t) = 2 u(x,t)$) grows with respect to *x*. When it decreases, there is the formation of a shock. When the shock is completely formed (purple color), the method correctly evolves the shock front. This technique allows overcoming the major limitation of the solution methods described in the previous section, easily ensuring mass-conservation of the various components, and accurately propagating the sharp front of the fluid component.

We also considered the Courant–Friedrichs–Lewy (CFL) restrictions in our simulations. In particular, at any fixed spatial resolution, one has to select a time step size so that the evolution is convergent in time. We checked that the used time step size at any resolution fulfills the CFL conditions. For example, in this test case of the Burgers’ equation, we set, to accurately follow the formation of the shock discontinuity the CFL factor to be 0.001.

### Linear waterflood

To verify that the numerical code is properly solving the governing equations and dealing with sharp front coming from discontinuities, it is important to compare our numerical results with analytical results from simplified examples. An analytical solution for a one-dimensional problem is given by the Buckley–Leverett model^44^ which estimates the advance of a fluid displacement front in an immiscible displacement process in the horizontal direction (zero gravity force) and zero capillary pressure.

The one-dimensional test is performed using full 3D-code evolution and imposing constant values of all variables along with two directions with constant boundary conditions achieving an effective 1D evolution. Along this direction is keeping fixed pressure difference and the evolved direction in *x* is $L= 25\,\mathrm{m}$ long with a cell resolution of $dx = 0.125\,\mathrm{m}$. We consider a pipe in which the initial pressure was set to be a fixed gradient. This is not a profile of a stationary two-phase fluid flow with constant flux. Nevertheless, in a few second it stabilizes to the right initial conditions. The data for this problem is given in Table 1. Relative permeabilities were taken from Eq. (15) as a function of water saturation.

**Table 1:** Data used in the numerical simulations to be compared with the one-dimensional Buckley–Leverett waterflood problem.

Parameter	Value
Absolute permeability, *k*	$4.14 \times 10^{-10}\,\mathrm{m}^2$
Porosity, $\phi _0$	0.30
Reservoir length, *L*	$25.00\,\mathrm{m}$
Cross-sectional area, *A*	$0.75 \times 0.75\,\mathrm{m}^2$
Oil viscosity, $\mu _n$	$0.10000\,\mathrm{kg}/\mathrm{m\,s}$
Water viscosity, $\mu _w$	$0.00100 \, \mathrm{kg}/\mathrm{m\,s}$
Oil density, $\rho _n$	$881 \, \mathrm{kg}/\mathrm{m}^3$
Water density, $\rho _w$	$1000 \, \mathrm{kg}/\mathrm{m}^3$
Van Genuchten, *n*, *m*	$n=2,m=1/2$
Irreducible wetting phase saturation, $S_{wir}$	0.057
Total pressure gradient	$\nabla p = 10^4 \, \mathrm{Pa}/\mathrm{m}$

**Figure 2:**
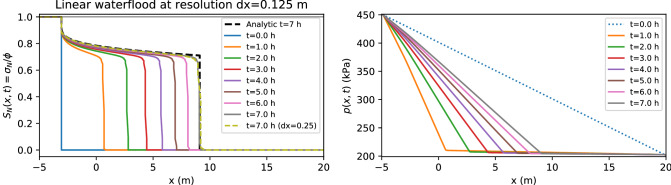
Comparison of NAPL saturation as a function of the distance from NAPL injection and the analytical solution^44^ and numerical results from CactusHydro code for a two-phase nonaqueous-water flood.

Figure 2, shows the behavior of the product of saturation and the porosity ($\sigma _{alpha}$) as a function of the distance for different values of the time, for a two-phase nonaqueous-water flood (with $\sigma _a=0$), and using CactusHydro code. Notice that $\sigma _a + \sigma _w + \sigma _n = \phi$ which is equal to 0.30 (see Table 1). We also show the comparison with a double resolution cell, $dx = 0.250\,\mathrm{m}$ which perfectly match with the one corresponsing to $dx = 0.125\,\mathrm{m}$. This also shows very good convergence for the results of the numerical simulations. We also compare the analytical result coming from the Buckley–Leverett model using the same parameters in the theoretical expression. Notice how the numerical results perfectly match the theoretical expectation even in the discontinuity zone. This is due to the use of a mass-conservative method implemented in the code. Relative permeabilities were chosen as a function of water saturation, as appears in Table 1, where the zero gravity and zero capillary pressure were imposed. From this Fig. 2 one can notice a sharp front predicted by the analytical solution and the numerical result which, fits perfectly.

**Figure 3:**
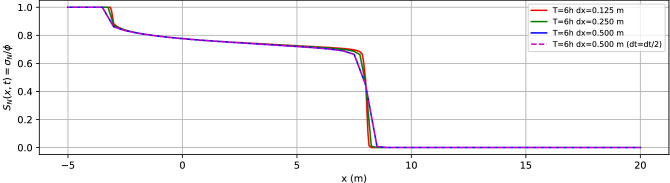
Comparison of NAPL saturation as a function of the distance from NAPL injection and the analytical solution^44^ and numerical results from CactusHydro code for a two-phase nonaqueous-water flood.

More in detail, Fig. 2 (left-hand side) showed the NAPL saturation as a function of the distance *x* from the NAPL injection at several values of *t* using a *dx* resolution of $0.125\,\mathrm{m}$. We then compared the analytical solution of the Buckley–Leverett model (the dashed black line at $t= 7.0$ h) with the numerical results at $dx=0.250\,\mathrm{m}$ (the cyan dashed line at $t=7.0$ h) and, $dx=0.125\,\mathrm{m}$ (the grey line at $t=7.0$ h). These lines fit very nicely. The pressure difference between the two end-point is kept fixed at the boundary, and indeed, the pressure gradient is changing with time as the nonaqueous component replaces water. See right-hand side of Fig. 2. Clearly, mesh-independency and time resolution should be checked. Fig. 3 shows (for the Buckley–Leverett problem) mesh-independency for several values of the spatial grid resolution ($dx=0.125\,\mathrm{m}, dx=0.250\,\mathrm{m}, dx=0.500\,\mathrm{m}$) where we use an equispaced cubic grid, and with respect to time resolution ($dt=dt/2$).

### Three-dimensional unsaturated three-phase fluid flow with gravity

An additional numerical test code, before showing a 3D example, is provided in the Supplementary Information. Our code results are compared with a two-dimensional numerical unsaturated–saturated water flow model^9^, together with the sand tank experimental data conducted by Koichi et al.^24^. See Table S1 for a list of parameters used in the 2D example, and Figure S1 for a comparison between the water table elevations measured in a sand tank experiment^24^ and the water table elevations calculated with CactusHydro as a function of the distance at different times..

Now that we have shown some tests, we can apply CactusHydro to a specific 3D example. In this example, we show the impact of an undetected leak of NAPL phase liquid onto a surface and, as a consequence of the gravity, moves from the unsaturated to the saturated zone. We consider a three-phase fluid case in which the contaminant is a light nonaqueous phase liquid (LNAPL) for which the density is less than the water. See Table 2 for details on the parameters. It provides a summary of the physical properties of the porous medium and fluids, initial conditions, and numerical model discretization data. The relative permeabilities and capillary pressure were taken from Eqs. (15, 19) which consider three-phase fluid flow relations.

**Table 2:** Data used in the 3D transient three-phase fluid flow, with an LNAPL released in the unsaturated porous medium zone that goes downward directed to the saturated aquifer. Data results from numerical simulations using CactusHydro.

Parameter	Value
Absolute permeability, *k*	$4.14 \times 10^{-10} \mathrm{m}^2$
Porosity, $\phi _0$	0.3
Simulated region , $x\times y \times z$	$80\,\mathrm{m}\times 20\,\mathrm{m} \times 30\,\mathrm{m}$
Resolution, *dx*	$0.50\,\mathrm{m}$
Oil viscosity, $\mu _n$	$10^{-1} \, \mathrm{kg}/\mathrm{m\,s}$
Water viscosity, $\mu _w$	$10^{-3} \, \mathrm{kg}/\mathrm{m\,s}$
Oil density, $\rho _n$	$881 \, \mathrm{kg}/\mathrm{m}^3$
Water density, $\rho _w$	$10^3 \, \mathrm{kg}/\mathrm{m}^3$
Van Genuchten, *n*, *m*	$n=2,m=1/2$
Irreducible wetting phase saturation, $S_{wir}$	0.057
Capillary pressure air-water at zero saturation, *pcaw*0	$0.081\,\mathrm{m}$
Capillary pressure air-nonaqueous at zero saturation, *pcan*0	$0.0566\,\mathrm{m}$

**Figure 4:**
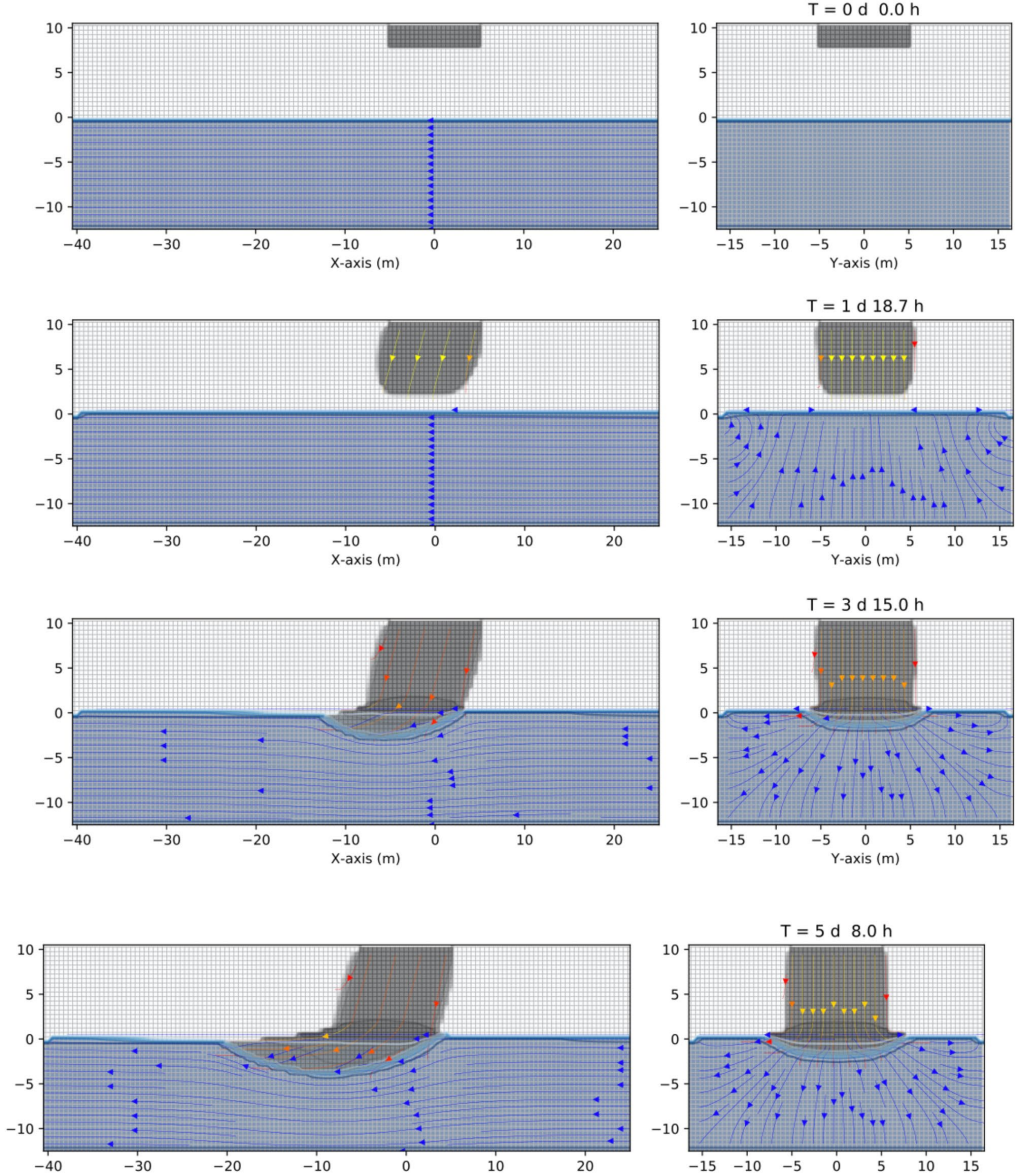
Example of a numerical simulation of a three-phase fluid flow simulation (water + LNAPL + air) performed using a spatial grid resolution of $0.50\,\mathrm{m}$ and a grid dimension of $80 \, \mathrm{m} \times 30\,\mathrm{m} \times 20\,\mathrm{m}$, at different times. The simulation has been performed using CactusHydro code. Left-hand side shows the (*z*–*x*) plane. Right-hand side shows the (*z*–*y*) one.

Figure 4 shows an example result of a numerical simulation of three-phase fluid flow (water, LNAPL, air) performed using a spatial grid resolution of $dx = 0.5\,\mathrm{m}$ and a total grid dimension of $80\,\mathrm{m} \times 30\,\mathrm{m}$ and $20\,\mathrm{m}$ in the z–x axis. The simulation has been successfully performed using CactusHydro on a parallel cluster dedicating to HPC at Parma University (Parma).

Initially, there is a constant leak of NAPL situated on top of this figure (left-hand side), in the unsaturated zone composed by a porous medium and a contaminant density situated at $z=10$ meters. The water table is at $z=0$ coordinate, and there is a gradient of gravity of 15 degrees. The LNAPL contaminant flows downward saturated zone due to the gravitational force. It then arrives at the water table, where a capillary pressure between air-contaminant and contaminant-water is present. The contaminant has a density lighter than the water. It remains around the water table while part of it goes in the left direction due to the pressure gradient (bottom, left-hand side). On the other column at the right-hand side, the same situation is viewed in the *z*–*y* plane. Notice the effect of the capillary pressure between contaminant and water, which causes a movement of the water flow in their vicinity. The right-hand side of this figure shows the *z*–*y* axis where there is a zero-gravity component effect and thus, no privileged direction in the *y* axes. The transient numerical simulation shows the behavior of this contaminant for a period of time of around five days, although it is possible to go further in the simulation.

## Conclusions

In this paper we have presented high-resolution 3D numerical results of three-phase immiscible fluids flow from the unsaturated to the saturated zone in a porous medium. They were obtained using a new code, CactusHydro, based on the Cactus toolkit (an open-source that provides a range of parallel computational capabilities). The governing equations are coupled general Richards’ equations for each phase (water, NAPL, and air). We investigated the three-phase immiscible fluid flow dynamics as a function of the saturation, capillary pressure, relative permeability of the different phases, using different initial and boundary conditions.

To deal with the sharp front originated from the partial differential equations’ nonlinearity and accurately propagate the fluid component sharp front, we use a high-resolution shock-capturing (HRSC) method to treat discontinuities due to capillary pressures and permeabilities that depend on the saturation of the three different phases. The innovative method considered in CactusHydro is the (first-order accurate) upwind fluxes in the conservative-flux methods discussed in^22^. This approach is based on (full) explicit in the time evolution of the discretized equation using the so-called methods-of-lines (MoL) for the time evolution of the discretized (in-space) variables, and a finite volume method MUSCL scheme (Monotonic Upstream-centered Scheme for Conservation Laws (van Leer, 1979).

We compare our numerical results with known analytical models such as the Burgers’ equation and the Buckley–Leverett model. An additional numerical test code is the comparison with a two-dimensional unsaturated–saturated water flow model, together with the sand tank experimental data conducted by Koichi et al.^24^ (see Supplementary Information). Lastly, we consider a simple three-phase fluid flow case in which a contaminant (LNAPL) leak onto the surface and, as a consequence of the gravity, moves from the unsaturated to the saturated zone.

These techniques allow overcoming the previous section’s solution methods’ major limitation and ensuring the various components’ mass-conservation. Also accurately propagating the sharp front of the fluid component. This has a drawback: the size of the time step that must be used to ensure convergence is much smaller than the one used with an implicit scheme or semi-implicit one like the one used in IMPES. The numerical results are very encouraging and show the robust capabilities of this code (and methods). We plan to apply it to more realistic cases in future works.

## Supplementary Information

**Supplementary Information.**
