# Supplementary material for: High-resolution shock-capturing numerical simulations of three-phase immiscible fluids from the unsaturated to the saturated zone
Source: Sci Rep. 2021 Mar 4;11:5212. doi: 10.1038/s41598-021-83956-w (PMC7933368; doi:10.1038/s41598-021-83956-w)
Supplement: Supplementary file 1 — Supplementary Information. [file 41598_2021_83956_MOESM1_ESM.docx]

**Supplementary Information**

**High-resolution Shock-Capturing Numerical Simulations of Three-Phase Immiscible Fluids from the Unsaturated to the Saturated Zone**

Alessandra Feo*^1,2^, Fulvio Celico^1^

^1^Department of Chemistry, Life Sciences and Environmental Sustainability, Parma University, Parco Area delle Scienze 157/A, 43124, Parma, Italy

^2^ INFN gruppo collegato di Parma, Parco Area delle Scienze, 7/A, 43124, Parma, Italy

*email: alessandra.feo@unipr.it

**Supplementary Table 1**

Table S1. List of parameters used for the two-dimensional (2D) example test code. CactusHydro is used to simulate a transient sand tank experiment conducted by Koichi et al. [29], and similar to a numerical test performed with FE-FDM and FEFLOW [16]. The sank tank is 3.15 m in length, 0.24 m in width, and 0.44 m in height. The boundary conditions are set to 0.20 m on the left and 0.00 m on the right, at $t=0.$ The top and bottom boundaries are impermeable. The initial condition is a water table at 0.00 m. For CactusHydro, we use a rectangular mesh with a resolution 0.04 m in each direction. CactusHydro, we use a rectangular mesh with a resolution of 0.04 m in each direction.

| Parameter | Value |
| --- | --- |
| Absolute permeability, $k$ | $3.36\times{10}^{-10} m^{2}$ |
| Porosity, $\phi_{0}$ | $0.44$ |
| Water viscosity, $\mu_{w}$ | ${10}^{-3} kg/\left( ms \right)$ |
| Water density, $\rho_{w}$ | ${10}^{3} kg/\left( ms \right)$ |
| Van Genuchten | $n=1.8, m=0.44$ |
| Irreducible wetting phase saturation, $S_{wir}$ | $0.091$ |
| $\alpha$ parameter | $34.48m^{-1}$ |
| Resolution | $0,04 m$ |

**Supplementary Figure 1**

Figure S1. Comparison between the water table elevations, measured in a sand tank experiment [29] (marked colored points), and the corresponding water table elevations computed with CactusHydro (continuum colored line) as a function of the distance, at different times. The numerical results fit very nicely with the experimental one for each time studied. The blue continuum line represents the boundary condition (at t=0 s.). The final line corresponds to a time where the system is already in the final state.
